# Stress-coping strategies and interpersonal characteristics in ineffective maternal adaptation after childbirth: a pilot case–control study

**DOI:** 10.3389/fpsyg.2026.1775660

**Published:** 2026-07-10

**Authors:** Jana Černá, Jiří Lenz, Ludek Fiala

**Affiliations:** 1 Faculty of Medicine in Pilsen, Charles University, Pilsen, Czechia; 2 Department of Clinical Psychology, University Hospital Pilsen, Pilsen, Czechia; 3 Department of Pathology, Znojmo Hospital, Znojmo, Czechia; 4 Department of Anatomy, Faculty of Veterinary Medicine, Veterinary University, Brno, Czechia; 5 Department of Sexology, University Hospital Pilsen, Pilsen, Czechia; 6 Institute of Sexology, First Faculty of Medicine, Charles University, Prague, Czechia

**Keywords:** adjustment disorder, interpersonal behavior, maternal mental health, postpartum adaptation, stress coping

## Abstract

**Background:**

Maternal adaptation after childbirth represents a complex psychological process that significantly influences maternal well-being, mother–infant interaction, and long-term child development. While postpartum depression and post-traumatic stress disorder have been extensively studied, less attention has been given to early stress-coping patterns and interpersonal characteristics associated with adjustment difficulties.

**Methods:**

This pilot case–control study included 104 women within the first postpartum year. Fifty seven women diagnosed with adjustment disorder (ICD-10: F43.2) formed the ineffective adaptation group, while 47 women without adaptation difficulties served as controls. Stresscoping strategies were assessed using the SVF 78 questionnaire, and interpersonal behavior was evaluated using the Interpersonal Checklist (ICL). Group differences were analyzed using non-parametric and categorical statistical tests.

**Results:**

Women with ineffective postpartum adaptation demonstrated significantly higher use of negative stress-coping strategies and lower use of positive strategies (all *p* < 0.001). Resignation, escape tendency, perseveration, and low positive self-instruction were significantly associated with ineffective adaptation. The accumulation of multiple maladaptive coping strategies was related to a substantially higher proportion of women experiencing adaptation difficulties. Interpersonal analysis indicated that a rebellious–distrustful personality pattern was significantly more frequent in the ineffective adaptation group.

**Conclusion:**

Ineffective postpartum adaptation, operationalized as adjustment disorder, is associated with specific maladaptive stress-coping strategies and interpersonal characteristics. These findings may contribute to improved clinical awareness and support the development of preventive psychosocial interventions in postpartum care.

## Introduction

Maternal adaptation after childbirth unfolds within a complex relational and social context. A mother interacts not only with her newborn but also with her partner, family members, and broader social environment. These interactions influence both her psychological adjustment and the quality of mother–infant bonding (Ashwal et al., 2019). Research on postpartum mental health has predominantly focused on depressive and posttraumatic symptomatology (Weis et al., 2020). However, adjustment disorder may represent an earlier and less severe manifestation of adaptation difficulties during the postpartum period. Understanding stress-coping strategies and interpersonal characteristics at this stage may provide insight into early maladaptive patterns before the development of more severe psychopathology (Terry et al., 1996). The present pilot case–control study examines stress-coping strategies and interpersonal characteristics associated with ineffective postpartum adaptation, operationalized as adjustment disorder (ICD-10: F43.2). By focusing on this early clinical level, the study aims to contribute to a more nuanced understanding of postpartum adaptation processes.

## Materials and methods

### Sample

The final sample comprised 104 women aged 20–43 years. Fifty-seven women diagnosed with adjustment disorder formed the ineffective adaptation group, while 47 women without adaptation difficulties served as controls.

Inclusion criteria were:

- Age between 20 and 43 years
- First postpartum year
- Ability to provide written informed consent
- Adjustment disorder (ICD-10: F43.2) was diagnosed by a licensed psychiatrist using a structured clinical interview based on ICD-10 criteria.

Exclusion criteria included:

- Major depressive disorder
- Bipolar disorder
- Psychotic disorders
- Substance use disorders
- Severe psychiatric conditions
- Severe neonatal medical complications

### Procedure

Participants were recruited from inpatient postpartum ward, outpatient clinics, and pediatric referrals. Assessments were conducted at 3, 6, or 9 months postpartum. The study protocol was approved by the Institutional Ethics Committee of the Faculty Hospital (No. 234/20). All participants provided written informed consent.

The study was conducted in accordance with the Declaration of Helsinki.

### Instruments

Stress-coping strategies were assessed using the SVF 78 questionnaire. Interpersonal characteristics were evaluated using the Interpersonal Checklist (ICL) (Leary et al., 1976).

### Statistical analysis

Descriptive statistics were calculated for all variables. Between-group differences were assessed using Wilcoxon rank-sum tests for continuous variables and χ^2^ or Fisher’s exact tests for categorical variables. Odds ratios were calculated where appropriate to estimate the strength of associations. Given the pilot nature of the study and moderate sample size, analyses focused on group comparisons rather than predictive modeling. Statistical significance was set at *p* < 0.05.

Women with ineffective postpartum adaptation demonstrated significantly lower median scores on positive coping strategies (EA median = 38; NEA median = 58; *p* < 0.001) and significantly higher median scores on negative coping strategies (EA median = 62; NEA median = 45; *p* < 0.001). Significant differences were observed for resignation, escape tendency, perseveration, and self-blame (all *p* < 0.001). Avoidance did not differ significantly between groups (*p* = 0.99). The proportion of women with ineffective adaptation increased with the number of elevated maladaptive coping strategies. Women presenting three or more identified risk factors were substantially more likely to belong to the ineffective adaptation group. Interpersonal analysis revealed that a rebellious–distrustful personality pattern was significantly associated with ineffective adaptation (χ^2^ = 9.806; *p* = 0.002).

## Discussion

The present pilot case–control study examined stress-coping strategies and interpersonal characteristics associated with ineffective postpartum adaptation operationalized as adjustment disorder. The findings indicate that women experiencing adjustment difficulties within the first postpartum year demonstrate a distinct psychological profile characterized by elevated maladaptive coping strategies and specific interpersonal tendencies.

### Maladaptive coping as a vulnerability marker

Women in the ineffective adaptation group reported significantly higher levels of resignation, perseveration, escape tendency, and self-blame, together with lower use of positive coping strategies. These findings are consistent with vulnerability–stress models suggesting that maladaptive cognitive-emotional processing styles may amplify the subjective impact of postpartum stressors. This interpretation is consistent with previous reviews highlighting the importance of coping processes in shaping psychological adjustment during the perinatal period (Guardino et al., 2014).

Perseveration, in particular, may reflect a tendency toward repetitive negative thinking resembling rumination. Repetitive cognitive fixation on stressors has been repeatedly associated with affective dysregulation and impaired problem-solving capacity. In the postpartum context–characterized by sleep deprivation, role transition, and heightened responsibility–such cognitive styles may interfere with adaptive maternal functioning and flexible coping (Nolen-Hoeksema et al., 2008).

Resignation and escape tendency may represent behavioral disengagement strategies that reduce active problem-solving and limit the utilization of available social support. Together, these coping patterns suggest not merely increased stress exposure, but altered stress processing.

### Adjustment disorder as an early stage of postpartum maladaptation

Most research in perinatal mental health has focused on major depressive disorder or posttraumatic stress disorder. The present study highlights adjustment disorder as a potentially earlier and clinically meaningful stage of maladaptation.

Although causal relationships cannot be inferred due to the cross-sectional design, the identified coping profile may represent an intermediate vulnerability pattern situated between normative adaptation stress and more severe psychopathology. From a developmental perspective, adjustment disorder could reflect an early manifestation of dysregulated stress adaptation mechanisms that, under persistent strain, may evolve toward more severe affective conditions.

This interpretation should be viewed as theoretical rather than predictive, yet it underscores the clinical relevance of detecting maladaptive coping patterns before the consolidation of more persistent psychopathology.

### Interpersonal characteristics and relational functioning

The finding that a rebellious–distrustful interpersonal pattern was significantly more frequent among women with ineffective adaptation adds a relational dimension to postpartum stress processing. Recent evidence also suggests that personality vulnerability and related biological markers, such as differential DNA methylation associated with depressive vulnerability, interact with resilience and affect perinatal mental health outcomes. For example, Castro et al. (2024) found associations between epigenetic markers, personality vulnerability to depression, and perinatal depressive symptoms, highlighting how underlying vulnerability constructs may shape postpartum psychological adaptation.

Within Leary’s interpersonal circumplex framework, this pattern reflects increased interpersonal defensiveness, autonomy sensitivity, and relational distrust. In the postpartum period–when effective help-seeking and reliance on social support are crucial–such interpersonal tendencies may reduce perceived support availability or hinder collaborative caregiving dynamics.

Interpersonal distrust may also contribute to reduced engagement with healthcare professionals, thereby delaying early psychosocial intervention. These findings suggest that postpartum adaptation is not solely an intrapsychic coping process but unfolds within an interpersonal regulatory context.

### Clinical implications

The results suggest that early screening for maladaptive coping strategies and interpersonal rigidity may represent a feasible preventive target within routine postpartum care. Brief psychological screening tools assessing perseverative thinking, resignation, and dysfunctional coping could complement traditional depression-focused screening approaches. Rather than viewing postpartum maladaptation exclusively through a depressive lens, incorporating stress-processing and interpersonal dimensions may allow earlier identification of women at risk (Sockol et al., 2013).

### Limitations and future directions

Several limitations warrant consideration. First, the study employed a cross-sectional case– control design, precluding causal inference. Second, the moderate sample size limits statistical power and generalizability. Third, participants were recruited from clinical settings, potentially introducing selection bias. Fourth, potentially relevant confounding variables– such as parity, socioeconomic status, and obstetric complications–were not systematically controlled.

Future research should employ longitudinal designs to clarify temporal relationships between coping patterns and postpartum adjustment trajectories. Larger samples would also allow multivariate modeling to evaluate independent predictors of maladaptation (Howard et al., 2014).

## Conclusion

This pilot case–control study suggests that ineffective postpartum adaptation, operationalized as adjustment disorder, is associated with a distinct profile of maladaptive stress-coping strategies and specific interpersonal characteristics. Elevated resignation, perseveration, escape tendency, and self-blame, together with reduced positive coping, were significantly more frequent among women experiencing adjustment difficulties.

Although causal conclusions cannot be drawn, the findings emphasize the potential importance of stress-processing styles and interpersonal functioning in early postpartum adjustment. Integrating these dimensions into routine postpartum psychological assessment may enhance early identification of women at risk and inform preventive psychosocial interventions. Further longitudinal research is required to determine whether these coping patterns represent transient adaptation phenomena or stable vulnerability markers.

## Data Availability

The original contributions presented in this study are included in this article/supplementary material, further inquiries can be directed to the corresponding author.
